# Integration of metabolomics and transcriptomics reveals novel biomarkers in the blood for tuberculosis diagnosis in children

**DOI:** 10.1038/s41598-020-75513-8

**Published:** 2020-11-11

**Authors:** Noton K. Dutta, Jeffrey A. Tornheim, Kiyoshi F. Fukutani, Mandar Paradkar, Rafael T. Tiburcio, Aarti Kinikar, Chhaya Valvi, Vandana Kulkarni, Neeta Pradhan, Shri Vijay Bala Yogendra Shivakumar, Anju Kagal, Akshay Gupte, Nikhil Gupte, Vidya Mave, Amita Gupta, Bruno B. Andrade, Petros C. Karakousis

**Affiliations:** 1 Center for Tuberculosis Research, Department of Medicine, Johns Hopkins University School of Medicine, 1551 East Jefferson Street, Room 110, Baltimore, MD 21287 USA; 2 Center for Clinical Global Health Education, Department of Medicine, Johns Hopkins University School of Medicine, Baltimore, MD USA; 3 Laboratório de Inflamação E Biomarcadores, Instituto Gonçalo Moniz, Fundação Oswaldo Cruz, Salvador, Brazil; 4 Multinational Organization Network Sponsoring Translational and Epidemiological Research (MONSTER) Initiative, Salvador, Brazil; 5 Curso de Medicina, Faculdade de Tecnologia E Ciências, Salvador, Brazil; 6 Byramjee Jeejeebhoy Government Medical College, Johns Hopkins University Clinical Research Site, Pune, Maharashtra India; 7 Byramjee Jeejeebhoy Government Medical College, Pune, Maharashtra India; 8 Johns Hopkins University - India Office (CCGHE), Pune, Maharashtra India; 9 Universidade Salvador (UNIFACS), Laureate Universities, Salvador, Brazil; 10 Escola Bahiana de Medicina E Saúde Pública (EBMSP), Salvador, Brazil; 11 Department of International Health, Johns Hopkins Bloomberg School of Public Health, Baltimore, MD USA

**Keywords:** Bacteria, Infectious-disease diagnostics, Pathogens, Systems biology, Biomarkers

## Abstract

Pediatric tuberculosis (TB) remains a major global health problem. Improved pediatric diagnostics using readily available biosources are urgently needed. We used liquid chromatography-mass spectrometry to analyze plasma metabolite profiles of Indian children with active TB (n = 16) and age- and sex-matched, *Mycobacterium tuberculosis*-exposed but uninfected household contacts (n = 32). Metabolomic data were integrated with whole blood transcriptomic data for each participant at diagnosis and throughout treatment for drug-susceptible TB. A decision tree algorithm identified 3 metabolites that correctly identified TB status at distinct times during treatment. N-acetylneuraminate achieved an area under the receiver operating characteristic curve (AUC) of 0.66 at diagnosis. Quinolinate achieved an AUC of 0.77 after 1 month of treatment, and pyridoxate achieved an AUC of 0.87 after successful treatment completion. A set of 4 metabolites (gamma-glutamylalanine, gamma-glutamylglycine, glutamine, and pyridoxate) identified treatment response with an AUC of 0.86. Pathway enrichment analyses of these metabolites and corresponding transcriptional data correlated N-acetylneuraminate with immunoregulatory interactions between lymphoid and non-lymphoid cells, and correlated pyridoxate with p53-regulated metabolic genes and mitochondrial translation. Our findings shed new light on metabolic dysregulation in children with TB and pave the way for new diagnostic and treatment response markers in pediatric TB.

## Introduction

The World Health Organization estimates incident tuberculosis (TB) occurs in 1.1 million children annually, representing > 200,000 deaths and 11% of global burden^1^. India ranks first in pediatric TB cases among 22 high-burden countries^2^. Since young children with pulmonary TB generally cannot cough effectively, microbiological confirmation of *Mycobacterium tuberculosis* (MTB) infection is established in only 15–50% of cases^2,3^, and often only after invasive sample collection^4^. Although rapid molecular tests have improved sensitivity over smear microscopy, their sensitivity in children remains < 70% compared with culture^5–7^. Furthermore, neither tuberculin skin tests (TST) nor interferon-gamma release assays (IGRA) can distinguish active disease from latent TB infection (LTBI)^8,9^. As a result, TB treatment is often prescribed empirically based on clinical findings and medical history. This inevitably over-diagnoses some children with associated toxicity and costs^10^.

Despite these diagnostic challenges, TB induces profound changes in whole-body energy and protein metabolism that can be identified in blood^11–21^. Such testing could also potentially identify extrapulmonary TB without invasive sampling of pleural or cerebrospinal fluid^22–24^ and improve assessments of treatment response^25–27^. Limited data are available, however, on the metabolic changes affecting children with TB^12,13^, and it is unknown whether the same differences described in other populations and body compartments will be relevant for blood-based diagnosis of pediatric TB or that the same metabolites will characterize treatment response among children. To characterize TB-associated metabolic dysregulation and assess potential blood-based biomarkers of TB and treatment response in children, we performed a longitudinal case–control study of Indian children with confirmed TB and uninfected household contacts.

## Material and methods

### Study design and sample selection

The Cohort for TB Research by the Indo-US Medical Partnership (CTRIUMPH) is a 5-year prospective observational study of adults and children with TB and the household contacts of participants with pulmonary TB^28^. The current analysis represents a nested case–control study in CTRIUMPH for which the methods have been described previously^29^. CTRIUMPH participants recruited at one of the sites, Byramjee Jeejeebhoy Government Medical College (BJGMC) in Pune, India with confirmed TB who were under 15 years of age were identified as cases, all of whom were successfully treated without clinical or microbiological relapse within 1 year of treatment completion. All participants received standardized therapy according to local standard of care in the public sector at the time of treatment (2015–2016). This included a thrice weekly fixed-dose drug combination of isoniazid, rifampin, pyrazinamide along with ethambutol. All drugs were dosed according to weight. All participants received all four drugs for the first two months of treatment, followed by a weight-based fixed dose combination of isoniazid and rifampin for the remainder of therapy (Table S1). Cases were each age- and sex-matched with two children who were household contacts of pulmonary TB patients (controls). Controls were considered eligible for this study if at the time of enrollment they had a negative symptom screen for TB, negative chest radiography, and both negative TST (< 5 mm induration) and IGRA (measured by QuantiFERON Gold In-Tube according to manufacturer’s instructions). Plasma was collected longitudinally from all cases at the time of treatment initiation, after 1 month of treatment, and at treatment completion at 6 months. Controls had plasma collected at enrollment and again at 4–6 months and 12 months, with repeat assessments at each time point for both active TB and LTBI, defined as positive by either TST or IGRA. Controls with new LTBI had the month of test conversion registered for analysis (Table S2).

### Quantitative metabolomic analysis

Samples were processed at Metabolon, Inc. (Durham, North Carolina) using the automated MicroLab STAR system from Hamilton Company and quality-control analyses were performed as described previously^30,31^. Briefly, several recovery internal standards were added prior to the first step in the extraction process for QC purposes. Samples were placed briefly on a TurboVap (Zymark) to remove the organic solvent. The sample extracts were stored overnight under nitrogen before preparation for analysis by ultrahigh performance liquid chromatography-tandem mass spectroscopy (UPLC-MS/MS). All methods utilized a Waters ACQUITY ultra-performance liquid chromatography (UPLC) and a Thermo Scientific Q-Exactive high resolution/accurate mass spectrometer interfaced with a heated electrospray ionization (HESI-II) source and Orbitrap mass analyzer operated at 35,000 mass resolution. The sample extract was dried and reconstituted in solvents compatible with each of the four methods particularly, to (1) optimize for more hydrophilic compounds, (2) analyze acidic positive ion conditions, (3) operate at an overall higher organic content, and (4) optimize basic negative ions. Each reconstitution solvent employed fixed concentration standards to ensure injection and chromatographic consistency. MS analysis alternated between MS and data-dependent MS^n^ scans using dynamic exclusion. The scan range varied slightly between methods but covered 70–1000 m/z. Raw data were extracted, peak-identified, and QC-processed using Metabolon’s hardware and software as described previously^32^. Compounds were identified by comparison to Metabolon library entries of purified standards or recurrent unknown entities.

### Transcriptomic data

Previous research in this cohort performed RNA-seq on longitudinal whole blood samples collected in PAXgene tubes^29^. Transcriptional signatures were identified for pediatric TB diagnosis and response to successful TB treatment with data publicly available from NCBI (accession code PRJNA588242). For this study, transcriptomic and metabolomic analyses used samples from the same participants and time points.

### Statistical analysis

Data were analyzed in R (https://cran.r-project.org). Differences in mean log_2_-transformed abundance between study groups were assessed by two-sample t-tests. ANOVA contrasts identified metabolites that differed significantly between groups and sample collection times. Data with p-values < 0.05 were considered significantly different. Exploratory analysis included visual inspection of volcano and principal component plots and construction of Venn diagrams of differentially abundant metabolites (DAMs, p < 0.05) across comparison groups. Log_2_-transformed metabolite abundance was Z-score-normalized between study groups and heatmaps were created with hierarchical clustering by Euclidean distance using ggplot2^33^. Random forest analysis was employed to estimate how well each metabolite correctly classified each sample and decision tree classification was performed to detect the minimum number of features required to separate groups with maximum accuracy using the rpart package^34^. Classification model performance was measured by receiver operator characteristic (ROC) curve analysis. Overall accuracy was estimated by area under the ROC curve (AUC).

We have previously defined the molecular degree of perturbation (MDP) as a metric that compares mRNA transcript expression or metabolite abundance to the mean of expression or abundance in the reference group^35,36^. The accumulated standard deviation of all metabolites > 2 absolute standard deviations from the reference were calculated using the MDP package^37^. MDPs were correlated with age by Spearman correlation test and MDP was compared by sex using Mann–Whitney tests. Enrichment analysis was performed to correlate metabolomic and transcriptomic data using a hypergeometric test for the main categories of metabolic compounds using the Clusterprofile^38^ and Reactome Pathway Analysis packages^39^. Hierarchical cluster analysis was performed using a log_2_-transformed expression matrix to visualize patterns by study group, with a second heatmap made to cluster metabolite expression levels against the increase of MDP measurement within each group^36^.

Integrated pathway analysis correlated metabolite abundance with transcript abundance expressed by protein-coding gene for each participant at each time point. Associations with p < 0.05 and Pearson coefficients > 0.7 were plotted to assess the extent to which DAMs were correlated with relevant mRNA transcripts. Multi-omics factor analysis (MOFA) was employed to determine the degree to which metabolite abundance and mRNA expression variables changed together, as assessed by shared variance using the MOFA package^40^. Variables with shared variance were represented as latent factors based on a common variable model. Finally, each factor underwent over-representation analysis to determine the extent to which latent factors represented distinct canonical Reactome pathways.

### Ethics statement

This CTRIUMPH study was approved by the Institutional Review Boards of Byramjee Jeejeebhoy Government Medical College (BJGMC) and the Johns Hopkins University School of Medicine, and all experiments were performed in accordance with institutional guidelines and regulations. All participants < 18 years old had written informed consent provided by their legal guardians. Written assent for participation was provided by participants who were 8 to < 18 years old.

## Results

### Study participants

Among the 141 pediatric CTRIUMPH participants recruited at BJGMC, 16 (11.3%) children had TB confirmed by positive culture (6), Xpert MTB/RIF (6), or tissue histopathology consistent with TB (7, not mutually exclusive). None were HIV-infected. The median age of these 16 children was 9.5 years (interquartile range 7–14 years), 8 (50%) were female, and eight had extrapulmonary disease (six lymph node, two meningitis, one dermatologic, Table S1). Of 32 selected controls without LTBI upon enrollment, 13 (40.6%) developed incident LTBI and none developed active TB during the 12-month follow-up period. These participants provided 121 plasma samples for analysis (Table S2).

### TB-associated changes in metabolites

**Figure 1:**
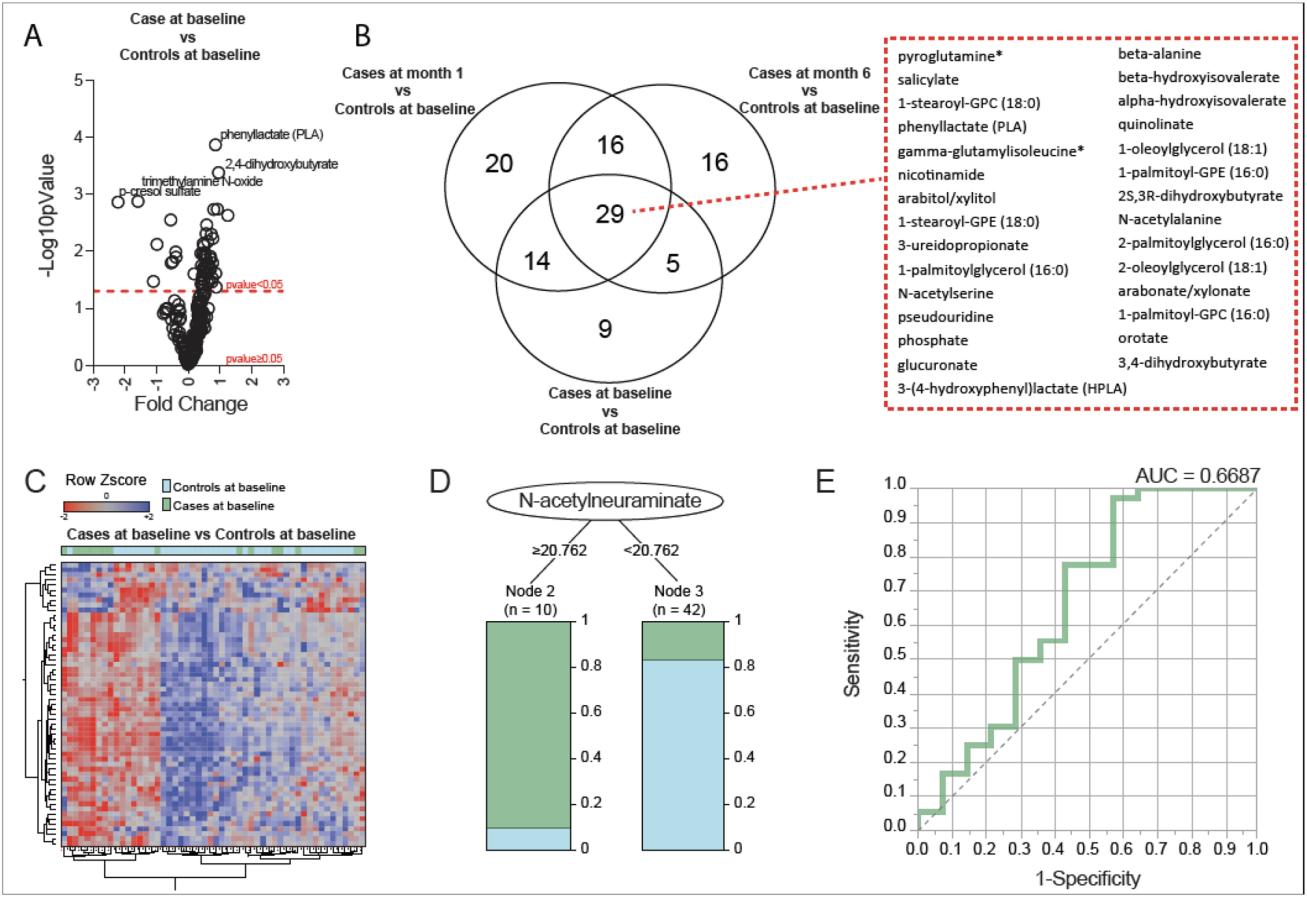
Differences in plasma metabolites between children with tuberculosis and healthy controls. (**A**) A volcano plot depicts the log_2_-fold change in metabolite abundance (x-axis) and the –log_10_ p-value (y-axis) for each metabolite between children with tuberculosis (“Cases”) and healthy controls at baseline. Positive and negative fold-change differences are depicted on the right and left sides of the graph, respectively. (**B**) A Venn diagram depicts the number of significantly differentially abundant metabolites for each comparison, including differences between controls and children with tuberculosis (cases) pre-treatment (baseline), mid-treatment (Month 1), and post-treatment (Month 6). (**C**) A hierarchical cluster analysis was employed to assess overall metabolite abundance between cases and controls. Data were log_2_-transformed and normalized by row Z-score. (**D**) A conditional decision tree was used to discriminate cases at the time of TB diagnosis from controls at study enrollment. The single best metabolite to differentiate cases and controls was N-acetylneuraminate. (**E**) Receiver operator characteristic (ROC) curve analysis demonstrating the sensitivity, specificity, and area under the curve (AUC) of N-acetylneuraminate to discriminate participants by TB status.

To study TB-associated metabolic dysregulation, we first compared plasma metabolite abundance among cases to abundance among controls at the time of enrollment. A total of 217 metabolites were identified across all samples, the full results of which are available in the supplementary materials (Table S3). Fifty seven DAMs were identified between enrollment samples (month 0) from cases and controls with p < 0.05 (Fig. 1A,B, Table 1). Of these, 29 DAMs were also differentially abundant between controls and cases mid-treatment (month 1) and between controls and cases at treatment completion (month 6), indicating continued metabolic abnormalities throughout treatment (Fig. 1B and Table 2). Additionally, 9 metabolites differentiated cases from controls at baseline (month 0), but were not differentially abundant between controls and cases after treatment (month 6). This included higher levels of 1-stearoyl-2-oleoyl-GPC (18:0/18:1), glutamate, and N-acetylneuraminate in cases compared to controls and lower levels of 1-(1-enyl-palmitoyl)-2-arachidonoyl-GPC, 1-(1-enyl-palmitoyl)-2-linoleoyl-GPC, aconitate [cis or trans], hydroxyproline, p-cresol sulfate, and prolylhydroxyproline in cases compared to controls. After evaluating heatmaps of differential abundance (Fig. 1C), decision tree analysis found that a single metabolite, N-acetylneuraminate, performed the best, with a log_2_-transformed abundance value < 20.76, achieving an AUC of 0.66 (Fig. 1D,E).

**Table 1:** Differentially expressed metabolites between children with confirmed tuberculosis and age- and sex-matched uninfected household contacts at the time of study enrolment.

1-(1-enyl-palmitoyl)-2-arachidonoyl-GPC (P-16:0/20:4)^a,b^	3-hydroxydecanoate^a^	Glucose	p-cresol sulfate^a,b^
1-(1-enyl-palmitoyl)-2-linoleoyl-GPC (P-16:0/18:2)^a,b^	3-ureidopropionate	Glucuronate	Phenyllactate (PLA)
1-oleoylglycerol (18:1)	Aconitate [cis or trans]^a,b^	Glutamate^b^	Phosphate
1-palmitoylglycerol (16:0)	Alanine	Hippurate^a^	Proline
1-palmitoyl-GPC (16:0)	Allantoin	Hydroxyproline^a,b^	Prolylhydroxyproline^a,b^
1-palmitoyl-GPE (16:0)	Alpha-hydroxyisovalerate	Kynurenine	Pseudouridine
1-stearoyl-2-oleoyl-GPC (18:0/18:1)^b^	Arabitol/xylitol	Lactate	Pyroglutamine
1-stearoyl-GPC (18:0)	Arabonate/xylonate	Mannitol/sorbitol	Quinolinate
1-stearoyl-GPE (18:0)	Azelate (nonanedioate; C9)	Mannose	Ribonate (ribonolactone)
2,4-dihydroxybutyrate	Beta-alanine	N-acetylalanine	Salicylate
2-oleoylglycerol (18:1)	Beta-hydroxyisovalerate	N-acetylglycine	Succinate
2-palmitoylglycerol (16:0)	Dihomolinolenate (20:3n3 or 3n6)	N-Acetylneuraminate^b^	Trimethylamine N-oxide^a^
2S,3R-dihydroxybutyrate	Erythronate	N-acetylserine	
3-(4-hydroxyphenyl)lactate (HPLA)	Gamma-glutamylisoleucine	Nicotinamide	
3,4-dihydroxybutyrate	Gamma-glutamylleucine	Orotate	

^a^Levels of these metabolites marked with footnote were decreased in the plasma of children with tuberculosis compared to age- and sex-matched controls without tuberculosis. Metabolites not marked with footnote were increased in the plasma of children with tuberculosis compared to those matched controls without tuberculosis.

^b^Footnote indicates metabolites that were differentially abundant between cases and controls at baseline, but not between controls and mid-treatment cases (Month 1) or post-treatment cases (Month 6).

**Table 2:** Differentially expressed metabolites between children with confirmed tuberculosis and age- and sex-matched uninfected household contacts that remained differentially expressed before, during, and after successful tuberculosis treatment.

1-oleoylglycerol (18:1)	2-oleoylglycerol (18:1)	Alpha-hydroxyisovalerate	Glucuronate	Phosphate
1-palmitoyl-GPC (16:0)	2-palmitoylglycerol (16:0)	Arabitol/xylitol	N-acetylalanine	Pseudouridine
1-palmitoyl-GPE (16:0)	2S,3R-dihydroxybutyrate	Arabonate/xylonate	N-acetylserine	Pyroglutamine
1-palmitoylglycerol (16:0)	3-(4-hydroxyphenyl)lactate (HPLA)	Beta-alanine	nicotinamide	Quinolinate
1-stearoyl-GPC (18:0)	3-ureidopropionate	Beta-hydroxyisovalerate	Orotate	Salicylate
1-stearoyl-GPE (18:0)	3,4-dihydroxybutyrate	Gamma-glutamylisoleucine	Phenyllactate (PLA)	

### Response to TB treatment

**Figure 2:**
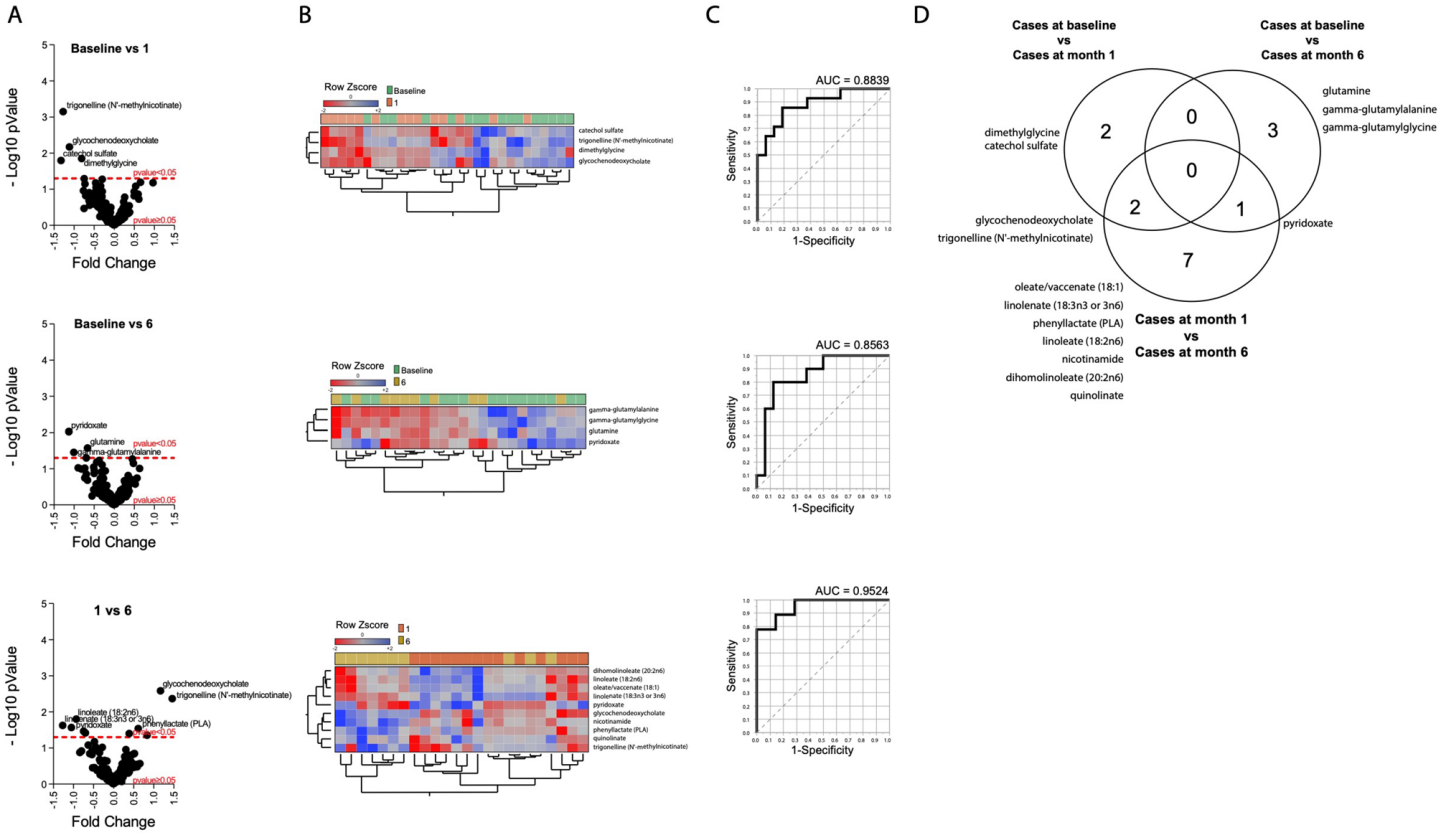
Plasma metabolic dysregulation during the treatment of TB in children. Differentially expressed metabolites between pre-treatment (month 0), mid-treatment (month 1), and post-treatment (month 6) samples collected from children with tuberculosis. (**A**) The volcano plots demonstrate the fold difference in metabolite abundance between study groups on the x-axis and the -log_10_ p-value of each difference on the y-axis. Extreme values on the x-axis indicate greater differences in abundance and the highest − log_10_ p-value represents the most significantly altered metabolite. The figures represent the differential abundance of metabolites between treatment months 0 and 1 (top), 0 and 6 (middle), and 1 and 6 (bottom). (**B**) Heatmaps of Z-score normalized metabolite plasma concentrations for the metabolites that were significantly differentially abundant between study groups. (**C**) Receiver Operator Characteristics (ROC) curve analysis for the combination of significantly differentially abundant metabolites differentiating children with TB at each time point during treatment. (**D**) The Venn diagram demonstrates the number of significantly differentially abundant metabolites across comparisons based on months of treatment.

Analysis of treatment response among cases compared metabolite abundance in pre-treatment (month 0), mid-treatment samples (month 1), and post-treatment samples (month 6), with heatmaps demonstrating distinct clustering but some misclassification (Fig. 2A,B). Multiparametric ROC curve analysis found 15 metabolites with differential abundance over the course of treatment (Fig. 2C). Four metabolites (catechol sulfate, dimethylglycine, glycochenodeoxycholate, and trigonelline (N’-methylnicotinate)) differentiated pre-treatment from mid-treatment samples at 1 month with an AUC of 0.88. Ten metabolites (dihomolinoleate (20:2n6), glycochenodeoxycholate, linoleate (18:2n6), linolenate (18:3n3 or 3n6), nicotinamide, oleate/vaccenate (18:1), phenyllactate (PLA), pyridoxate, quinolinate, and trigonelline (N'-methylnicotinate)) differentiated mid-treatment (month 1) from post-treatment (month 6) with an AUC of 0.95, and 4 metabolites (gamma-glutamylalanine, gamma-glutamylglycine, glutamine, and pyridoxate) differentiated pre-treatment from post-treatment samples with an AUC of 0.86 (Fig. 2D).

Conditional decision tree analysis identified three distinct metabolites that best classified patients with TB at different times during treatment compared to controls at enrolment: N-acetylneuraminate, quinolinate, and pyridoxate (Figure S2). In addition to the characteristics of N-acetylneuraminate described above, quinolinate was found to have an AUC of 0.77 (threshold log_2_-transformed abundance of < 18.07) for identification of TB following 1 month of treatment compared to controls at baseline, and pyridoxate was found to have an AUC of 0.87 (threshold log_2_-transformed abundance of < 19.21) for identification of TB at the completion of 6 months of treatment compared to controls at baseline. Although it maintained good discriminating ability, pyridoxate was not one of the DAMs between cases and controls at baseline (p = 0.076) or between cases later in treatment and controls at baseline (pre-treatment vs. end of treatment p = 0.009, mid-treatment vs. end of treatment p = 0.016).

### Molecular degree of perturbation (MDP) is independent of participant age or sex

**Figure 3:**
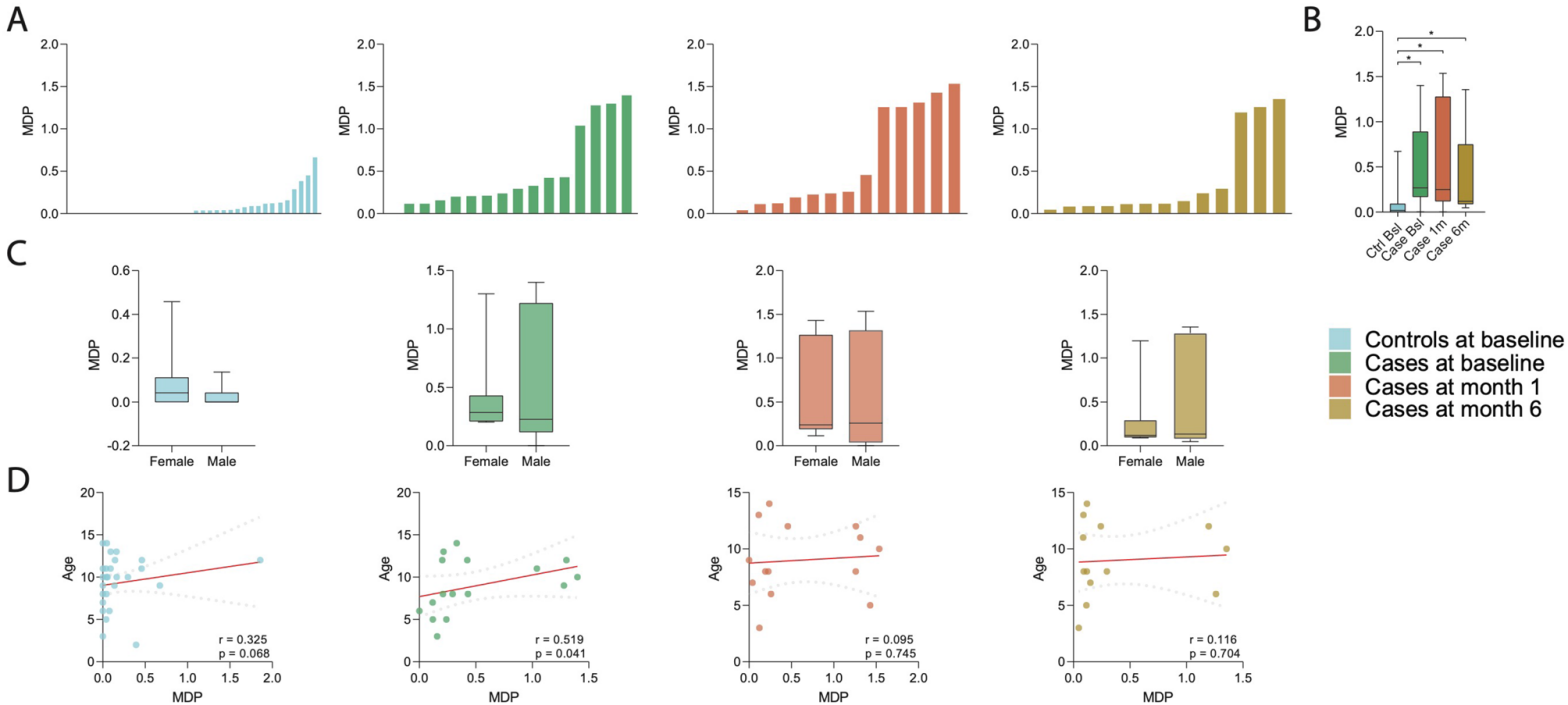
Molecular degree of perturbation among children with tuberculosis during treatment and uninfected controls, by participant age and sex. (**A**) Histograms show the single sample molecular degree of perturbation (MDP) score values for controls at baseline (blue), cases before treatment (green), cases after 1 month of treatment (orange), and TB cases after 6 months of treatment (dark yellow). (**B**) Out of these groups, MDP was significantly higher among cases before treatment (green) and cases after 1 month of treatment (orange) than among controls. MDP was not significantly different between cases after treatment (yellow) and controls (blue). (**C**) MDP was not significantly different by sex in any study group. (**D**) MDP was positively correlated with increasing age of participant among cases before treatment, but not among other study groups.

MDP was calculated for each sample to investigate overall differences in metabolite concentrations compared to baseline control samples. Plasma metabolites from TB cases at month 1 of treatment (orange) were the most perturbed, followed by the TB cases at baseline (green, Fig. 3A,B). MDP was not different by sex for any study group (Fig. 3C), but increasing MDP was significantly correlated with increasing age among cases at baseline (r = 0.519, p = 0.041, Fig. 3D). While study groups demonstrated significant differences according to MDP, this was poorly correlated with metabolic variance among individual participants within study groups, indicating poor representation of metabolite abundance in these children by MDP alone (Figure S1).

### Integrated analysis of metabolomic and transcriptomic data

**Figure 4:**
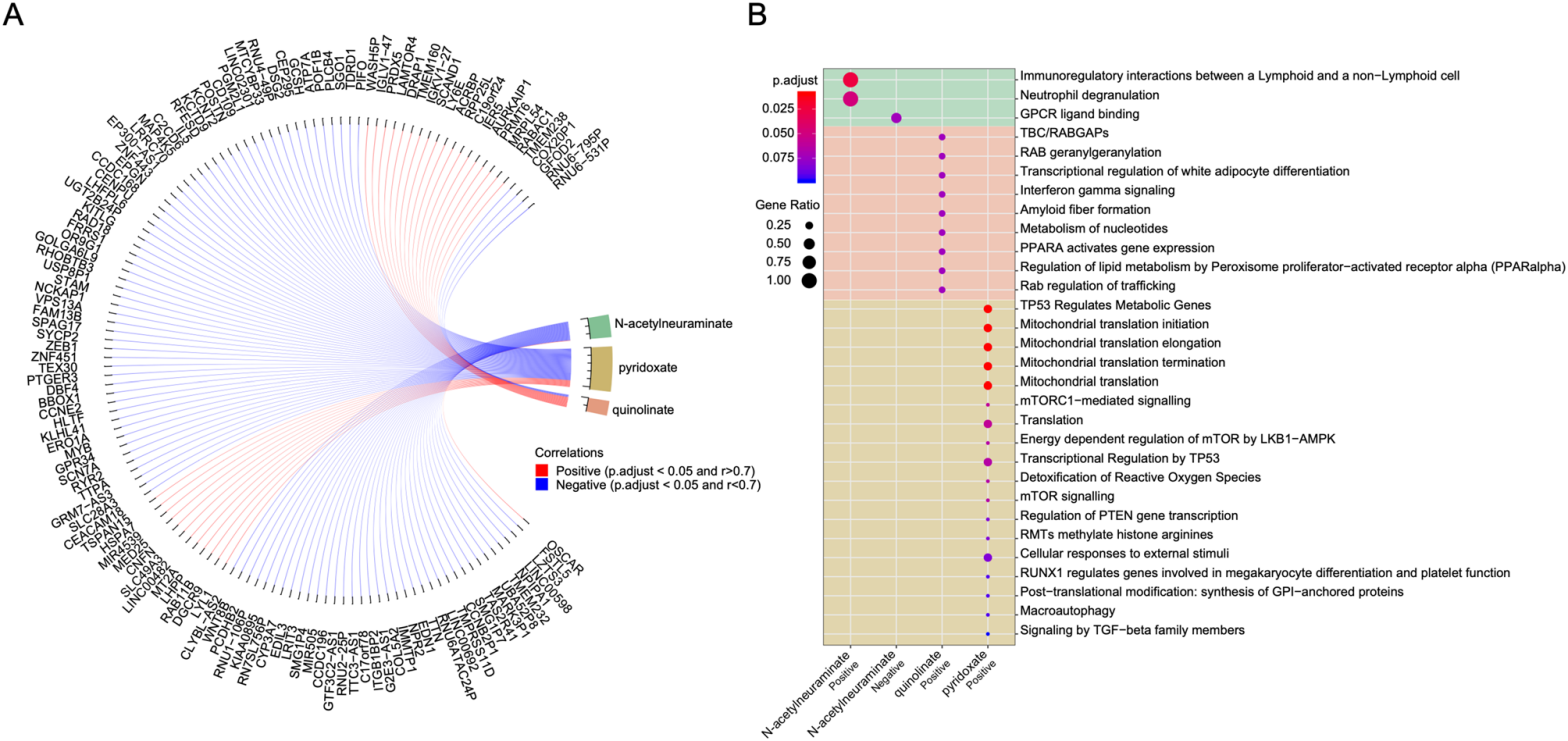
Integration of metabolomics and transcriptomics data reveals a complex and multifaceted immune response to TB. (**A**) Correlation network based on gene expression values in TB cases. Highlighted genes were found to correlate with N-acetylneuraminate, quinolinate and pyridoxate with p-value < 0.05 and absolute value of R > 0.7. (**B**) List of pathways associated with genes found to be positively and negatively correlated with the above-mentioned 3 metabolites.

Three metabolites (N-acetylneuraminate, quinolinate, and pyridoxate) identified by decision tree analysis were correlated with mRNA transcription data to assess the extent to which metabolite abundance increased with increased gene transcription (positive correlation, Fig. 4A) or decreased with increased gene transcription (negative correlation). In total, 22 transcripts demonstrated strong correlation with N-acetylneuraminate, (1 positively correlated and 21 negatively correlated). The top positive and negatively correlated genes for N-acetylneuraminate were osteoclast-associated receptor (*OSCAR*) and CLYBL, respectively. Transcripts positively correlated with N-acetylneuraminate at the time of TB diagnosis were significantly represented among the pathway involving lymphoid immunoregulatory interactions (Fig. 4B). Similarly, 72 transcripts demonstrated strong correlation with quinolinate (12 positive and 60 negative correlations) and 22 transcripts with pyridoxate (18 positive and 4 negative correlations). The genes positively correlated with quinolinate did not adhere to any specific pathway, but those correlated with pyridoxate were significantly represented in the TP53-regulated metabolic pathway and in mitochondrial translation (elongation, termination, and translation). Transcripts with negative correlation with these metabolites did not cluster with any specific pathways.

### Multi‐omics factor analysis (MOFA) of metabolomic and transcriptomic data

**Figure 5:**
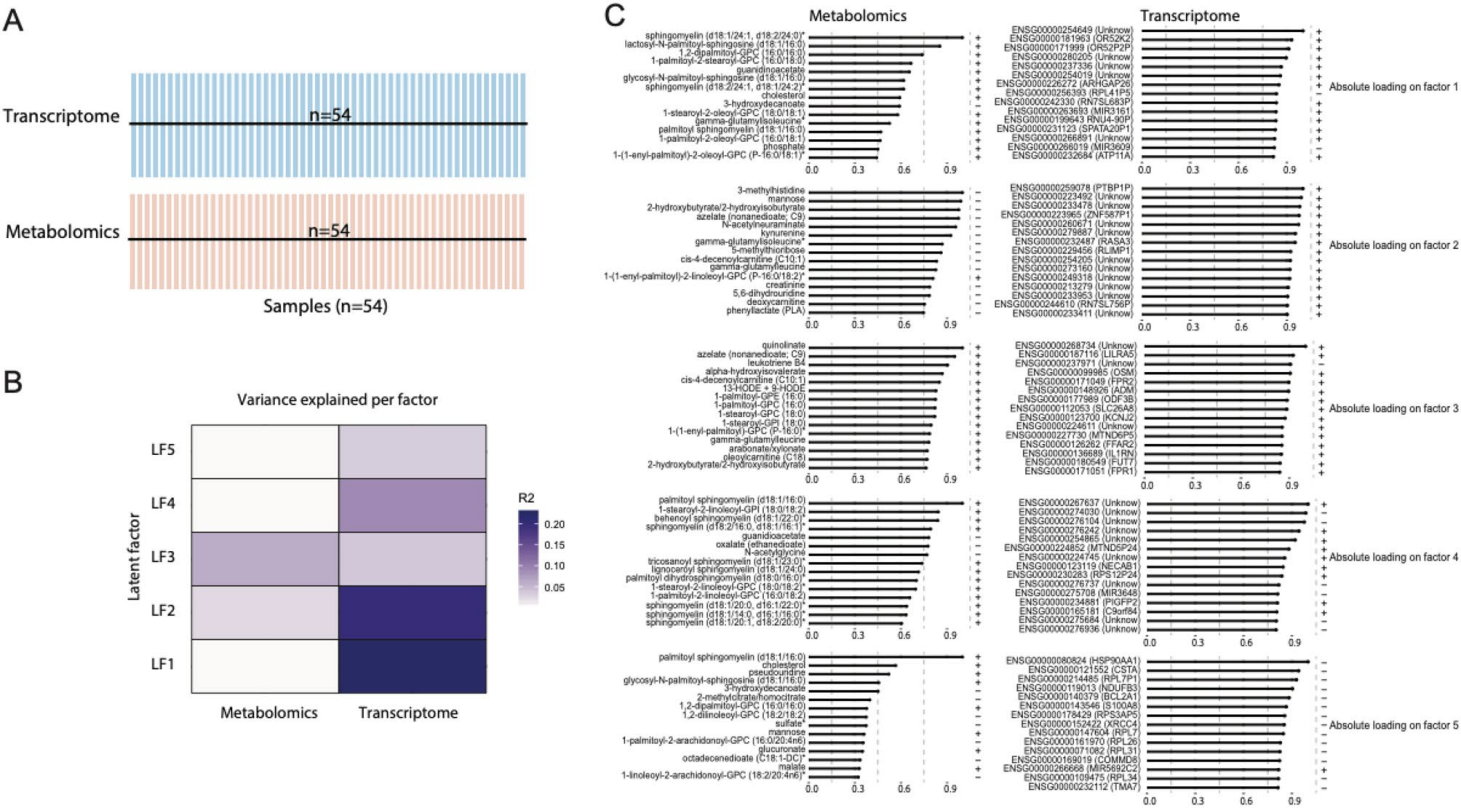
Downstream Multi‐Omics Factor Analysis (MOFA) disentangles the variability between metabolomics and transcriptomics data. The fitted MOFA model assessed the proportion of variance explained by each factor in each data modality. (**A**) Study overview and data types. Data modalities are shown in different rows (d = number of features) and samples (n) in columns. (**B**) The proportion of total variance (R^2^) explained by individual factors for each assay. (**C**) Absolute loadings of the top features of Factors 1 to 5 in the metabolomics (left panel) and transcriptomics (right panel) data.

Integrated metabolomic and transcriptomic analysis by MOFA was applied to 54 paired samples from the same participants and collection times (Fig. 5A). MOFA recognized 5 latent factors (LF), of which LF1, LF2, and LF4 correlated more strongly with the transcriptome, whereas LF3 correlated with the metabolome (Fig. 5B). Transcriptomics and metabolomics loadings revealed that LF1–5 captured several known important genes related to immune response pathways, including leukocyte immunoglobulin-like receptors (LILR), olfactory receptor 52K2, NADH-ubiquinone oxidoreductase chain 4 (MT-ND4), and heat shock protein 90α (Hsp90α) encoded genes. Similarly, several important metabolites were identified, including sphingolipids, 3-methylhistidine (3-MH), quinolinate, and palmitoyl sphingomyelin (Fig. 5C). Over-representation analysis of each LF found eukaryotic translation elongation, metabolism of proteins, immune system, and gene expression pathways most significantly represented within the 5 latent factors.

## Discussion

In order to improve the management and outcomes of pediatric TB, better diagnostic tools are desperately needed. In 2014, an NIH expert panel developed a blueprint of diagnostic biomarkers for pediatric TB^7^. According to the panel, such a biomarker could be obtained without invasive testing, like a blood test, and would work independent of age, nutrition, and HIV status. In this study, we performed an integrated blood-based metabolomic and transcriptomic analysis of children with and without TB to identify such a biomarker to indicate TB diagnosis and treatment response. We identified 57 DAMs between cases and controls, 29 of which remained differentially abundant throughout treatment, and 9 of which were differentially abundant before treatment, but did not remain differentially abundant throughout treatment. A single metabolite, N-acetylneuraminate, identified TB with an AUC of 0.66, while another metabolite, quinolinate, discriminated mid-treatment TB samples from control samples (AUC 0.77), and a third, pyridoxate, best discriminated post-treatment samples from control samples (AUC 0.87). The combination of 4 metabolites classified pre-treatment from post-treatment samples with an AUC of 0.86. Integrative analysis associated N-acetylneuraminate with mRNA transcription in the lymphoid immunoregulatory and neutrophil degranulation pathways. Finally, MOFA confirmed a significant additional contribution of immunomodulatory gene transcription along with sphingolipids, histidine metabolites, and cofactor and vitamin metabolites.

This study is unique in its metabolomic profiling of children with confirmed TB (rather than clinically diagnosed TB), as well as the use of decision tree analysis and integration of metabolomic with transcriptomic data to identify the most high-yield tests for both diagnosis and response to antitubercular treatment.

TB affects multiple host metabolic pathways, including metabolism of nitric oxide^14^, amino acids such as tryptophan^15,17,18,25,41^, glucose^17^, and lipids, including sphingomyelins, ceramides, eicosanoids, and phosphotidylcholines^13,16,42^. This study found potential diagnostic utility of N-acetyleneuraminate, the main sialic acid in human and mammalian cells, as well as a a nutrient and cell surface component of many pathogenic bacteria^43,44^. This molecule has previously been reported in higher abundance among TB patients, but not as a single marker for the diagnosis of TB^18^. Similarly, we found quinolinate to be the best marker to differentiate TB mid-treatment from controls. Quinolinate is a byproduct of the kynurenine pathway responsible for tryptophan catabolism, and is primarily derived from microglia and macrophages. Tryptophan metabolism has been associated with both pulmonary and extrapulmonary TB, as well as with HIV coinfection, which, like pediatric TB, is more frequently associated with extrapulmonary disease^22,24^. In addition, quinolinate levels are increased in a range of bacterial and viral infections^45,46^, as the inflammatory cytokines IFN-γ and TNF-α shuttle tryptophan from serotonin towards the kynurenine pathway^47^. We found quinolinate to differentiate mid-treatment cases from controls with an AUC of 0.77, which is lower than published data finding quinolinate to identify TB at the time of diagnosis (AUC = 0.84)^16^. This may reflect changes in inflammation and metabolism during the early phase of treatment, as quinolinate rose mid-treatment before falling at the end of treatment in our data (Table S3). Finally, pyridoxate was the metabolite that best differentiated cases following treatment completion from controls with an AUC of 0.87. Pyridoxate, a breakdown product of B6, is a potential biomarker for TB due to its role in human cellular immunity and the ability of MTB to consume host B6^20^. This suggests that the differential abundance between TB patients post-treatment and controls may reflect resolving infection, although follow-up studies are needed to confirm this hypothesis.

This is not the first attempt to combine metabolites into a diagnostic signature for TB. Previous studies have achieved AUC values of 0.86–0.99 among adults, including 0.84 among people with HIV^16,25,42^. Pediatric studies are less common, however, with one study achieving AUC values between 0.76–0.81 using a variety of techniques, and another achieving an AUC of 0.98, representing better sensitivity of metabolomic signatures in this population than smear microscopy, Xpert MTB/RIF, or urine lipoarabinomannan testing^12,13^. The simplest metabolic biomarker we identified for TB, N-acetyleneuraminate, achieved an AUC of 0.66, which, while lower than values achieved in studies of adults with TB, is similar to the AUC of urine lipoarabinomannan (LAM) in children (AUC 0.67) and better than that of LAM among children without advanced HIV (AUC 0.46)^48^. Our data also found that the combination of 4 metabolites (gamma-glutamylalanine, gamma-glutamylglycine, glutamine, and pyridoxate) identified an initial treatment response with an AUC of 0.86, which—if confirmed in other studies including patients who fail treatment—represents improvement over smear and culture conversion to predict durable cure, which have only 24% and 40% sensitivity, respectively^49^. Previous studies have identified metabolomic signatures of treatment response, including one study identifying a 4 metabolite signature with 86.2% sensitivity, 85.2% specificity and an AUC of 0.91^27^, and another study finding changes of 5 metabolites during treatment^50^. These specific metabolites were not significantly different between pre- and post-treatment samples in our study, demonstrating the need to replicate diagnostic accuracy studies in special populations, such as children, before they are broadly employed.

The integrative analysis in this study did not support the addition of MDP to metabolomic assessment to improve diagnostic yield. Nevertheless, it did confirm the correlation of TB-induced metabolic dysregulation with lymphoid and non-lymphoid immunoregulation, as well as with mitochondrial translation. MOFA found strong correlations with transcriptomic and metabolomic data. In our previous work, we have defined transcriptional signatures of pediatric TB and treatment response, achieving AUCs for diagnosis of 0.57–0.90 and AUCs for treatment response of 0.50–0.90 in external datasets^29^. This suggests that there is room to improve diagnostic accuracy of metabolic assessments by incorporating multiple modalities, as in the current work.

This study has several limitations. While we have focused on an assessment of children with confirmed TB, these results may not be generalizable to adults, who represent the majority of TB patients worldwide. Similarly, TB has a well-known interaction with HIV coinfection, which was not assessed in this study population. While this study was designed to assess metabolic changes among children with confirmed TB, it is important to consider that the majority of children, particularly infants, treated for TB do not have confirmed disease. The choice to study children with confirmed TB likely raised the median age of study participants compared to clinically diagnosed pediatric TB patients^2,3^. Each of these may have contributed to distinct metabolic changes identified in our study population compared to other study populations.

In this single-site study of children with confirmed TB and exposed, uninfected household contacts of TB patients, we identified N-acetylneuraminate, quinolinate, and pyridoxate as candidate biomarkers for TB disease activity in children. The combination of 4 metabolites (gamma-glutamylalanine, gamma-glutamylglycine, glutamine, and pyridoxate) accurately identified treatment response. Integration of metabolomic and trancriptomic data from the same patients confirmed the involvement of several inflammatory pathways and improved interpretation of these biomarkers. Future validation studies will be needed to confirm these findings among other pediatric populations, including those coinfected with HIV. If successful, these tools may assist in the development of novel host-directed therapies for TB, as well as the use of predictors of treatment response to help shorten TB treatment. This would be particularly helpful for drug-resistant TB and for extrapulmonary TB, for which repeat microbiological assessment is often not possible during treatment.

## Supplementary information

Supplementary Information 1.

Supplementary Information 2.

## Data Availability

Data supporting the findings of this study are available within the article and its supplementary files, or are available from the authors upon request. Transcriptomics data analyzed in this manuscript are available from the NCBI sequence read archive (accession code PRJNA588242).
