# Supplementary material for: Integration of metabolomics and transcriptomics reveals novel biomarkers in the blood for tuberculosis diagnosis in children
Source: Sci Rep. 2020 Nov 11;10:19527. doi: 10.1038/s41598-020-75513-8 (PMC7658223; doi:10.1038/s41598-020-75513-8)
Supplement: Supplementary file 1 — Supplementary Information 1. [file 41598_2020_75513_MOESM1_ESM.docx]

**Integration of metabolomics and transcriptomics reveals novel biomarkers in the blood for tuberculosis diagnosis in children**

Noton K. Dutta^+1*^, Jeffrey A. Tornheim^+1,2^, Kiyoshi F. Fukutani^∂3,4,5^, Mandar Paradkar^∂6^, Rafael T. Tiburcio^4,5^, Aarti Kinikar^7^, Chhaya Valvi^7^, Vandana Kulkarni^6^, Neeta Pradhan^6^, Shri Vijay Bala Yogendra Shivakumar^8^, Anju Kagal^7^, Akshay Gupte^2^, Nikhil Gupte^2,6^, Vidya Mave^2,6^, Amita Gupta^1,2,6,11^, Bruno B. Andrade ^#4,5,6,9,10^, and Petros C. Karakousis^#1,11*^ for the CTRIUMPH RePORT India Study Team

**Affiliations**:

^1^Center for Tuberculosis Research, Department of Medicine, Johns Hopkins University School of Medicine, Baltimore, MD, USA

^2^Center for Clinical Global Health Education, Department of Medicine, Johns Hopkins University School of Medicine, Baltimore, MD, USA

^3^Laboratório de Inflamação e Biomarcadores, Instituto Gonçalo Moniz, Fundação Oswaldo Cruz, Salvador, Brazil.

^4^Multinational Organization Network Sponsoring Translational and Epidemiological Research (MONSTER) Initiatine, Salvador, Brazil.

^5^Curso de Medicina, Faculdade de Tecnologia e Ciências, Salvador, Brazil.

^6^Byramjee Jeejeebhoy Government Medical College – Johns Hopkins University Clinical Research Site, Pune, Maharashtra, India

^7^Byramjee Jeejeebhoy Government Medical College, Pune, Maharashtra, India

^8^Johns Hopkins University – India office (CCGHE), Pune, Maharashtra, India

^9^Universidade Salvador (UNIFACS), Laureate Universities, Salvador, Brazil

^10^Escola Bahiana de Medicina e Saúde Pública (EBMSP), Salvador, Brazil

^11^Department of International Health, Johns Hopkins Bloomberg School of Public Health, Baltimore, MD, USA

^+,∂,#^ These authors contributed equally

**Supplementary Table S1: List of study participants with tuberculosis, including site of extrapulmonary disease, diagnostic test results, and treatment received.**

| Site of Tuberculosis | Extrapulmonary Site | AFB Smear Result | Xpert MTB/RIF Result | Culture Result | Histopathology Result | Treatment Regimen and Duration |
| --- | --- | --- | --- | --- | --- | --- |
| Pulmonary | Not applicable | Positive | Positive | Positive | Not applicable | 2HRZE + 5HR |
| Pulmonary | Not applicable | Negative | Negative | Positive | Not applicable | 2HRZE + 4HR |
| Pulmonary | Not applicable | Positive | Positive | Positive | Not applicable | 2HRZE + 4HR |
| Pulmonary | Not applicable | Positive | Positive | Positive | Not applicable | 2HRZE + 4HR |
| Pulmonary | Not applicable | Negative | Positive | Positive | Not applicable | 2HRZE + 4HR |
| Pulmonary | Not applicable | Negative | Positive | Positive | Not applicable | 2HRZE (Off Study, Not Included in Treatment Response Analysis) |
| Pulmonary | Not applicable | Negative | Negative | Positive | Not applicable | 2HRZE (Off Study, Not Included in Treatment Response Analysis) |
| Pulmonary and Extrapulmonary | Lymph node | Negative | Negative | Negative | Positive | 2HRZE + 4HR |
| Extrapulmonary | Meninges | Negative | Positive | Positive | Not applicable | 2HRZE + 10HR |
| Extrapulmonary | Skin | Negative | Negative | Negative | Positive | 2HRZE + 8HR |
| Extrapulmonary | Lymph node | Negative | Negative | Negative | Positive | 2HRZE + 4HR |
| Extrapulmonary | Lymph node | Negative | Negative | Negative | Positive | 2HRZE + 4HR |
| Extrapulmonary | Lymph node | Negative | Negative | Negative | Positive | 2HRZE + 4HR |
| Extrapulmonary | Lymph node | Negative | Negative | Negative | Positive | 2HRZE + 4HR |
| Extrapulmonary | Lymph node | Negative | Negative | Negative | Positive | 2HRZE + 4HR |
| Extrapulmonary | Meninges | Negative | Positive | Negative | Not applicable | 2HRZE + 13HR |

All cases were diagnosed with tuberculosis through the combination of mycobacterial culture, Xpert MTB/RIF (“GeneXpert”), and histopathology results. All participants received standard therapy and had clinical and microbiological resolution of disease during the interval of study analysis. Two participants with culture positive pulmonary TB did not participate in the study for the full duration and were not included in the 6-month treatment response analysis.

**Supplementary Table S2: Study samples analyzed for global metabolic profiles in children with tuberculosis (“Cases”) and age- and sex-matched healthy controls, by sample collection month, N=121**


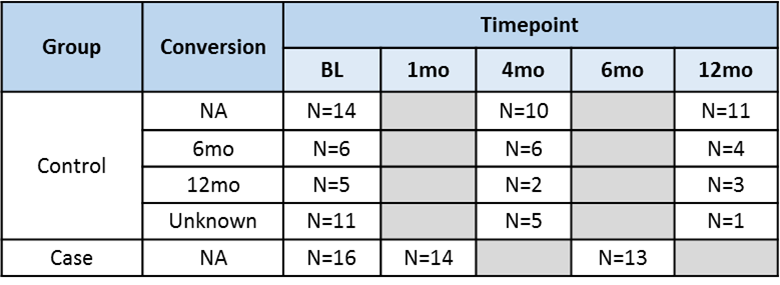


Each case was matched by age and sex to two household members of the participants with active TB. Controls were TST/ IGRA negative at enrollment. However, a few converted TST/IGRA or both at 4 / 12monts follow up visits. N= numbers, mo=months

**Supplemental Table S3: Plasma metabolites identified in cases and controls**

See attached excel file

Pubchem: Pubchem database (<https://pubchem.ncbi.nlm.nih.gov/>); KEGG: Kyoto Encyclopedia of Genes and Genomes (<https://www.genome.jp/kegg/>); HMDB: The Human Metabolome Database (<https://hmdb.ca/>); CAS: Chemical Abstracts Service Registry (<https://www.cas.org/support/documentation/chemical-substances>); RI: retention index

**Supplemental Figure S1. Heatmap of metabolite abundance among children with (“Case”) and without (“Ctrl”) tuberculosis by month of sample collection**


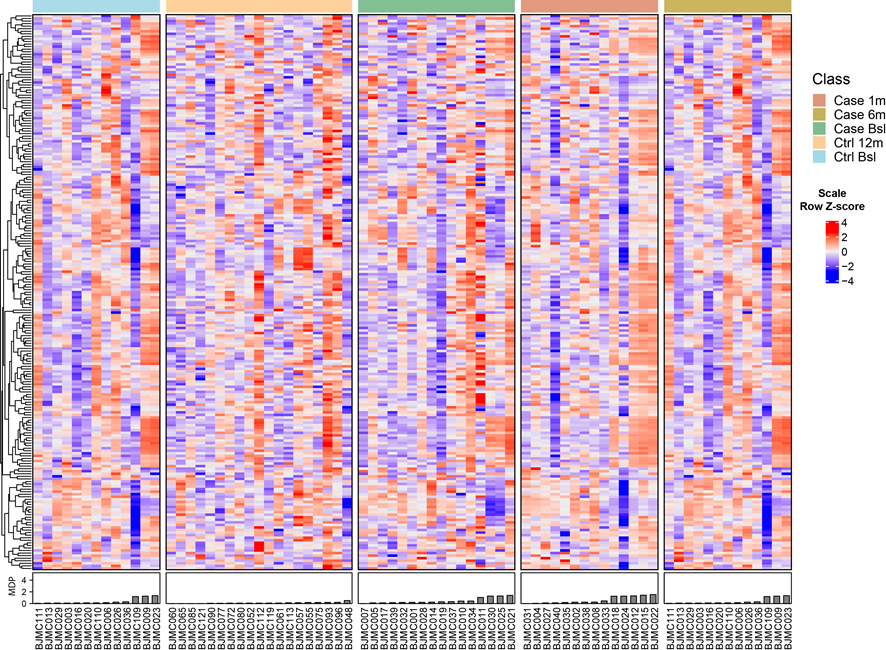


Rows are clustered by Euclidean distance between normalized row Z-score while columns were ordered by molecular degree of perturbation (“MDP”) within each study group. Metabolite abundance clustered poorly by MDP within each study group.

**Supplemental Figure S2.** **Conditional decision tree analysis for metabolites between children with tuberculosis during (“Case 1”) and after (“Case 6”) successful treatment compared to healthy controls at the time of enrolment (“Ctrl BSL”)**


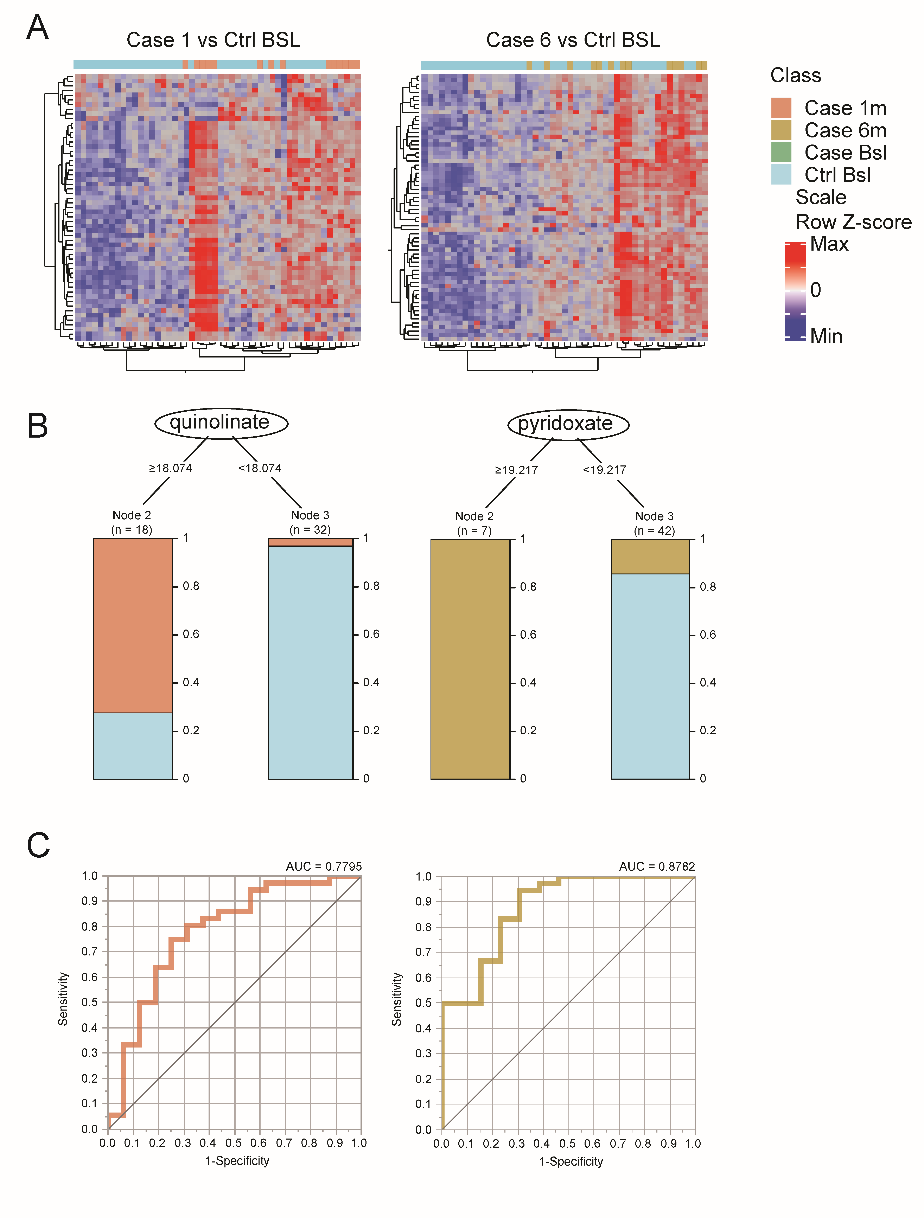


A) Metabolite plasma concentration values in each heatmap were normalized by Row Z-score, and clustered in columns and rows (two-way hierarchical cluster using Ward’s method). B) Conditional decision tree analysis to discriminate children with tuberculosis at each time point from healthy controls. Analysis identified the best metabolites that could classify children with TB at each time point from controls, which were quinolinate and pyridoxate for each time point, respectively. C) Receiver operator characteristic curves were constructed for each metabolite, with areas under the curve (“AUCs”) of 0.77 and 0.87 at 1 month and 6 months of successful treatment, respectively.

**Supplemental Acknowledgements**

The CTRIUMPH Study Team includes Aarti Kinikar, Alamelu Raja, Amita Nagraj, B Anand Kumar, Anita More, Archana Gaikwad, Ashwini Nangude, S Balaji, Beena Thomas, Bency Joseph, TK Bharath, B Brindha, Chhaya Valvi, David Dowdy, Deepak Pole, A Devanathan, M Devi Sangamithrai, Divyashri Jain, CK Dolla, Gabriela Smit, R Gangadarsharma, Hanumant Chaugule, Hari Koli, Hemanth kumar, J Jeeva, Jessica Elf, Jonathan Golub, Jyoti Chandane, Kanade Savita, M Kannan, M Karthikesh, S Karunakaran, Kelly Dooley, Lakshmi Murali, M Lavanya, Luke Hanna, S Madasamy, M Mageshkumar, S Mangaiyarkarasi, Mahesh Gujare, S Manoharan, M Michel Premkumar, P Munivardhan, S Murugesan, Nagaraj, C Ponnuraja, N Premkumar, Rahul Lokhande, S Rajkumar, K Ranganathan, S Rani, Renu Madewar, Robert Bollinger, Rosemarie Warlick, Rupak Shivakoti, Sahadev Javanjal, P Sathyamurthi, Shalini Pawar, Shashank Hande, Shital Muley, Shital Sali, K Shubhapriya, Shyam Biswal, K Silambu Chelvi, Smita Nimkar, Sriram Selvaraj, Sundeep Salvi, Swapnil Raskar, Uma Devi, Vandana Kulkarni, Vidula Hulyalkar, Vinod Tayawade, Vrinda Bansode, and Yogesh Daware.
